# Regulation of human endometrial function: mechanisms relevant to uterine bleeding

**DOI:** 10.1186/1477-7827-4-S1-S5

**Published:** 2006-10-09

**Authors:** Hilary OD Critchley, Rodney W Kelly, David T Baird, Robert M Brenner

**Affiliations:** 1 Department of Reproductive and Developmental Sciences, University of Edinburgh, Centre for Reproductive Biology, The Queen's Medical Research Institute, 47 Little France Crescent, Edinburgh EH16 4TJ, UK; 2 Medical Research Council Human Reproductive Sciences Unit, Centre for Reproductive Biology, The Queen's Medical Research Institute, 47 Little France Crescent, Edinburgh EH16 4TJ, UK; 3 Oregon National Primate Research Center, 505 NW 185^th ^Avenue, Beaverton, OR 97006, USA

## Abstract

This review focuses on the complex events that occur in the endometrium after progesterone is withdrawn (or blocked) and menstrual bleeding ensues. A detailed understanding of these local mechanisms will enhance our knowledge of disturbed endometrial/uterine function – including problems with excessively heavy menstrual bleeding, endometriosis and breakthrough bleeding with progestin only contraception. The development of novel strategies to manage these clinically significant problems depends on such new understanding as does the development of new contraceptives which avoid the endometrial side effect of breakthrough bleeding.

## Introduction

The uterus plays a pivotal role in the key events of primate reproduction, implantation, and in the absence of pregnancy, menstruation. Preparation of the endometrium for implantation is a consequence of exposure of this steroid target tissue to estradiol and progesterone [1,2]. Absence of pregnancy, demise of the corpus luteum and the subsequent fall in circulating progesterone leads to degradation and shedding of the superficial layer of the endometrium. Menstruation is a process of tissue injury and subsequent repair involving a complex interplay between the endocrine and local immune system [3,4]. It is well known that progesterone is the steroid critical for endometrial differentiation, but the specific determinants of endometrial receptivity and the complex molecular and cellular interactions involved are matters of current research. Modern, post-genomic technologies are being used to advance our knowledge of the complex events of implantation and menstruation [5-9].

Repetitive episodes of menstrual bleeding are the outward indicators of cyclical ovarian function. In the past, when contraception was not available or widely practiced large family sizes were associated with long periods of lactation which in turn suppressed ovulation and induced amenorrhea. As a consequence, women's lives were practically menstruation free [10,11]. Nowadays, because contraception is more widely practiced in developed societies, women may menstruate on the order of 400 times during their reproductive lifespan. Disorders of the menstrual process are therefore one of the banes of modern women.

The following review focuses on the complex events that occur in the endometrium after progesterone is withdrawn (or blocked) and menstrual bleeding ensues. A detailed understanding of these local mechanisms will enhance our knowledge of disturbed endometrial/uterine function – including problems with excessively heavy menstrual bleeding, endometriosis, and breakthrough bleeding with progestin only contraception. The development of novel strategies to manage these clinically significant problems depends on such new understanding as does the development of new contraceptives which avoid the endometrial side effect of breakthrough bleeding.

### Endometrial sex steroid receptor expression

#### 1. Endometrial progesterone receptor (PR) expression

Steroid hormones are the systemic factors that drive the endometrium through the characteristic sequential phases of the menstrual cycle [12]. Steroids interact with their target cells via specific nuclear receptors with consequential initiation of gene transcription and a cascade of downstream molecular and cellular events. Members of the nuclear receptor super-family expressed by endometrial cells include progestin, estrogen, androgen and glucocorticoid receptors. Estrogen receptor (ER) and progesterone receptor (PR) expression is under dual control of estradiol and progesterone, and along with the androgen receptor (AR) varies both temporally and spatially across the menstrual cycle [13-17].

Two isoforms of the human PR have been described [18,19]. PRA is the shorter isoform, missing 164 amino acids at the end terminus of the B subtype. PRA and PRB derive from a single gene and function as transcriptional regulators of progestin-responsive genes. There is a significant decline in PR expression in the glands of the functional layer of the endometrium with the transition from the proliferative to the secretory phase of the cycle. PR persists in the stroma in the upper functional zone, and is particularly highly expressed in the stromal cells in close proximity to the uterine vasculature. The basal region is regulated differently in that the glands and stroma of the deeper zones express PR throughout the cycle. Localisation studies utilising antibodies that recognise both PR isoforms have shown that PR is also differentially regulated in stromal versus epithelial cells. For example, in the secretory phase the PRB isoform declines in the stroma and PRA becomes the dominant form, while both forms decline in the epithelial cells of the functional layer [20,21]. The differential expression of the PR in the superficial and basal regions of the endometrium is likely to have functional importance because only the superficial layers are shed during menstruation.

The progesterone receptor has been described as being present in blood vessel walls but absent from the vascular endothelium of the human uterus [16,22]. In the rhesus macaque endometrium, PR is absent from both the endothelial cells of capillaries and the smooth muscle cells of the artery walls but is strongly expressed in the perivascular stroma, which is closely apposed to the muscular wall [23]. Therefore, the effects of progesterone or progesterone withdrawal on vasoconstriction and endometrial bleeding are likely to be indirect effects, mediated by the PR-positive perivascular stromal cells.

#### 2. Endometrial ER expression

Several of the fundamental processes involved in normal endometrial function, such as proliferation [24] and vascularization [25] are regulated by estrogen. Estrogen upregulates key endometrial genes including PR, vascular endothelial growth factor (VEGF), and lactoferrin [26-28]. Upregulation of PR in the proliferative phase is consistent with identification of estrogen response elements in the regulatory region of the PR gene [29] thus providing evidence for a functional estrogen mediated pathway in the proliferative phase. VEGF is a key local mediator of cyclical neovascularisation in the functional layer and VEGF mRNA increases in the mid proliferative phase [30].

Estrogen action is mediated by two variants of estrogen receptor referred to as ERα and ERβ [16,31-33]. The function of ERβ in the uterus is still to be determined. In the upper functional layer, ERα expression increases in both glandular and stromal cells in the proliferative phase and declines in the secretory phase due to suppression by progesterone. In the basal layer, ERα is expressed in glandular and stromal cells throughout the menstrual cycle in women [13,15] and nonhuman primates [34].

In human and non human primate endometrium [16,22,35,36], ERβ was the only sex steroid receptor that was present in the endothelium and smooth muscle walls of endometrial vessels. ERα, PR, and AR were not detectable in either the endothelium or vascular smooth muscle cells of primate endometrial vessels but were strongly expressed in the perivascular stroma where they were increased by estrogen and suppressed by progesterone in the functional layer. We concluded that any direct effects of estrogen on endometrial vessels, including angiogenesis and permeability, would be mediated by ER β, and that the actions of progestins and androgens would be mediated indirectly by perivascular stromal cells.

In vitro studies have demonstrated that homodimers (ERα- ERα or ERβ-ERβ) or heterodimers (ERα- ERβ) can be formed when both isoforms are expressed in the same cell [37,38]. When tested in vitro on a luciferase reporter gene linked to an estrogen response element and activated with estradiol or diethylstilbestrol, both receptor homodimers induce similar *trans*-activation profiles, but they signal in opposite ways at an activating protein-1 site [39]. Studies reported by Hall and McDonnell [40] have indicated that one role played by ERβ may be to modulate ERα transcriptional activity. A novel human ERβ variant named hERβcx (ERβcx/β2) formed by alternative splicing of the eighth exon of ERβ (ERβ1) has been identified [41]. Thus the structure of an estrogenic ligand, its concentration, the presence of co-regulators, and the subtype(s) of ER expressed in each cell will determine the pattern of estrogen-induced gene expression [42]. The cellular distributions of ERβ1 and ERβcx/β2 variants may have important implications for the regulation of implantation and menstruation, and also for dysfunctional aspects of these events. It has been reported that mRNAs corresponding to both ERβ1 and ERβcx/β2 variants were detectable in human endometrial tissue [36]. ERβcx/β2 variant was detected with a semi-quantitative PCR approach in all endometrial tissue samples regardless of the stage of the cycle. ERβ1 and ERβcx/β2 proteins were localized to multiple cell types within the endometrium through the use of isoform-specific monoclonal antibodies; immunoexpression of ERβ1 appeared more intense than that of ERβcx/β2 in endometrial epithelium and endothelial cells. Immunoexpression of ERβ1 appeared unchanged throughout the menstrual cycle. In contrast, levels of ERβcx/β2-specific immunoreactivity were specifically reduced in gland cells within the functional layer, but not in those of the basal layer, in the mid-secretory phase. Therefore, co-expression of ERβcx/β2 in cells containing ERβ1 and/or ERα could modulate the effects of estrogens on the endometrium [36].

#### 3. Endometrial androgen receptor (AR) expression

Exogenous androgen administration has an inhibitory effect on the human and non-human primate endometrium, and can induce endometrial atrophy. Elevated levels of circulating endogenous androgen have been described in a sub group of women with recurrent miscarriage implicating an antagonistic role for androgens on endometrial estrogen effects [43]. It has been demonstrated that androgens can suppress estrogen and progestin receptors in the reproductive tract of estrogen-treated, ovariectomized macaques [44]. Androgen receptor expression in human and non-human primate endometrium has been described [17,45]. In the macaque, ligand binding, immunohistochemistry (IHC) and in situ hybridisation (ISH) were used to demonstrate the regulation and localisation of AR during the hormonally regulated cycle. In women, endometrial androgen receptor expression was studied by IHC, ISH, and quantitative RT-PCR (Q-RT-PCR). During the normal cycle, in both women and macaques, stromal cells are the predominant cell type that express the AR. Androgen effects in the endometrium are thus likely mediated by the stroma. Further, endometrial AR levels are augmented by estradiol and suppressed by progesterone. Most interestingly, anti-progestin administration to either women or macaques enhances AR expression in the stroma and induces AR expression in the glands of the endometrium [17,46].

The physiological role of endometrial AR is yet to be determined. Androgens are known to suppress estrogen-dependent endometrial proliferation, and it has been sugested [47,48] that the endometrial AR mediates the anti-proliferative effects induced by anti-progestins. Furthermore, in the rhesus macaque, administration of the anti-androgen, flutamide, has been shown to counteract the suppressive effects produced by anti-progestins on endometrial thickness, stromal compaction and mitotic index [49]. In women and non-human primates, treatment with progesterone-antagonists suppresses estrogen-dependent mitotic activity in the endometrial glands. This anti-proliferative effect is paradoxical, because progesterone antagonists do not bind to the estrogen receptor. While this phenomenon has been termed a "functional noncompetitive antiestrogenic effect" [50] it does not occur in all species or in all regions of the primate reproductive tract, and hence is best referred to as an "endometrial antiproliferative effect" [48]. The endometrial androgen receptor may be a critical component of the mechanism by which anti-progestins suppress endometrial proliferation in the presence of circulating estrogens [48].

#### 4. Endometrial intracrinology

In human endometrium, steroidal regulation of receptor action depends on ligand availability. The 17βHSD enzyme family comprises eight members, of which six human isoforms have been characterised. The primary role of 17βHSD type2 is the conversion of estradiol to estrone as well as testosterone to androstenedione, but in addition it converts the inactive 20α-dihydroprogesterone to active progesterone [51,52]. There is good evidence that progesterone upregulates 17βHSD2 during the secretory phase [53,54] in the endometrial glandular epithelium [55]. Its activity decreases when progesterone concentrations decrease (as with luteal regression) or after anti-progestin administration [54,55].

Levonorgestrel (LNG) is a synthetic progestin that binds to both the AR and PR [56,57] and can increase the expression of 17βHSD2 protein, but this effect diminishes over time. For example, administration of LNG locally, by insertion of an intrauterine delivery system (IUS) in women, induced a dramatic transformation of the endometrium characterised by extensive decidualisation followed by slow atrophy over the long term [58-60]. There were high levels of endometrial 17βHSD2 protein identified in the first month after insertion of the LNG-IUS accompanied by high levels of 17βHSD2 mRNA for three months post insertion. After 3 months of treatment there was a decrease in 17βHSD2 protein in glandular epithelium and in total 17βHSD2 mRNA that persisted for up to 12 months. These shifts were associated with a down regulation of the PR over the same time frame [45].

**Figure 1:**
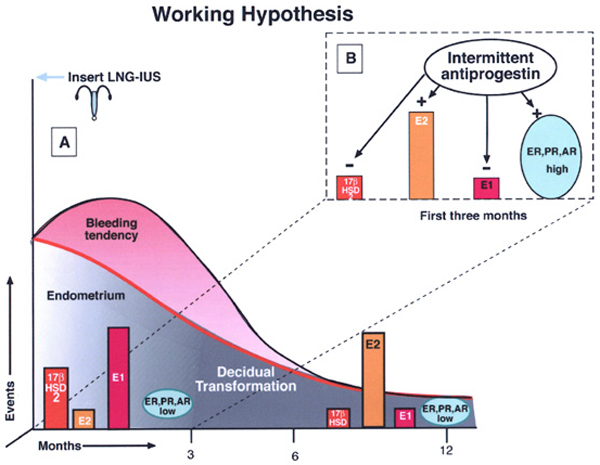
Endometrial changes after insertion of an LNG-IUS; the effects of intermittent antiprogestin therapy on bleeding. A working hypothesis. A Insertion of an LNG-IUS induces a process of decidualisation (grey colour) in the endometrium. During the first 3 months bleeding is high then slowly improves. During this time, 17βHSD-2 is elevated and therefore estrone, E_1 _(a less potent estrogen) is high and estradiol, E_2 _(the more potent estrogen) is low; steroid receptors are suppressed. After 6 months, when decidualisation is extensive, bleeding is reduced, 17βHSD-2 is low, E_2 _is high and E_1 _is low. Steroid receptors remain low, (though ERβ is likely to be present in the vascular endothelium). This balance of steroids at 6 months is associated with reduced bleeding. B. Intermittent antiprogestin treatment during the first 3 months will suppress 17βHSD-2 and thereby lower E_1_, elevate the more potent E_2_and elevate steroid receptors. This balance of steroids is similar to the balance at 6 months, but would allow E_2 _to interact with higher levels of receptors in all cell types and should suppress BTB. In addition, the spiral arteries would be strongly inhibited by antiprogestins, which should also suppress BTB. This periodic "putting on the brakes" on bleeding may help women through the most difficult period of adjustment to an LNG-IUS, after which antiprogestin treatment could be stopped.

Since 17βHSD2 converts estradiol to the less potent estrogen, estrone, the endometrial glands are exposed to more estrone than estradiol during the first 3 months after LNG-IUS insertion when the enzyme is elevated. Any estradiol-dependent products of the glands that have paracrine actions throughout the endometrium would thus be suppressed. It is during the first months after LNG-IUS insertion that a proportion of users report unscheduled breakthrough bleeding (BTB). We propose that the unscheduled BTB reported may be due in part to an intracellular "functional estrogen deficiency" caused by the elevated levels of 17βHSD2 that either directly or indirectly leads to vascular fragility. 17βHSD2 protein expression is significantly reduced following 3 months treatment, coincident with the onset in improvement in menstrual bleeding patterns. 17βHSD2 mRNA expression also declines, but this occurs more slowly, becoming undetectable after 6 months of LNG-IUS treatment. One way to control this pattern of BTB in women wearing an LNG-IUS may be to treat them intermittently with a progesterone antagonist. These compounds would increase endometrial estrogen receptor levels and lower 17βHSD2 expression thereby reversing any intracellular estrogen deficiency. Figure 1 summarizes our working hypothesis of a "functional estrogen deficiency" and illustrates the potential of intermittent treatment with progesterone antagonists to suppress BTB.

#### 5. Progesterone withdrawal and up regulation of matrix metalloproteinases (MMPs) and the VEGF receptor type 2 (kinase domain receptor -KDR)

During the secretory phase, the expression and activity of MMPs in endometrial stromal/decidual cells are inhibited by the circulating levels of progesterone. Sex steroid withdrawal at the end of the cycle reverses the inhibition of many MMPs, including MMP-1, which can degrade the interstitial collagens of the extracellular matrix (ECM) surrounding pre-decidual cells, especially in the superficial endometrium. The increase and activation of MMPs are considered key elements of the menstruation process [61-63]. There is also evidence that the focal expression of MMPs within peri-menstrual and menstrual endometrium involves local regulatory factors. Leukocytes can directly release MMPs and there are important interactions between endometrial leucocytes, stromal and epithelial cells which result in induction and activation of MMPs [64].

Data from rhesus macaques that have been treated with estradiol and progesterone indicate that the induction of menstruation and up-regulation of various MMPs is identical when either progesterone is withdrawn and estradiol is maintained, or when both estradiol and progesterone are withdrawn [2,65]. Moreover, the administration of the anti-progestin mifepristone (RU 486), during the mid secretory phase, is associated with marked endometrial ECM breakdown and excessive menstrual bleeding [66,67]. MMP-2 is one of the putative menstrual proteinases and there is evidence that stromal cell derived MMP-2 increases just before menstruation and participates in the breakdown of ECM. In vitro data clearly show that progesterone withdrawal up-regulates MMP-2 [68]. In summary, it is the withdrawal of progesterone, not estrogen, that is the essential signal which initiates upregulation of the MMPs, even though both steroids decline together at the demise of the corpus luteum.

Many other factors are also operative during the pre-menstrual phase [69-71]. For example, the VEGF receptor type 2 (KDR) is expressed primarily by vascular endothelium and only rarely in other cell types. However, KDR is dramatically up-regulated in the stromal cells of the superficial endometrial zones during the pre-menstrual phase in both human and macaque endometrium [72]. The up-regulation of stromal KDR follows progesterone withdrawal in both women and macaques, and adding back progesterone 24 hours after progesterone withdrawal in the macaque interrupted stromal, but not vascular, endothelial KDR expression. ProMMP-1 is also up-regulated in a coordinate manner in the same stromal cell population by progesterone withdrawal. Increases in endometrial vascular endothelial growth factor (VEGF) occur in the premenstrual phase, and, as discussed below, this factor may play a role through KDR in endometrial MMP expression and activation.

#### 6. Effects of vasoconstriction and presumed hypoxia

Transplantation of explants of endometrium to the anterior chamber of the eye of the rhesus macaque allowed Markee [1] to demonstrate that vasoconstriction of the spiral arteries was induced by the withdrawal of progesterone. The constriction of spiral arteries was associated with the regression of stromal tissue and onset of degradation of the ECM [1]. Hence progesterone withdrawal not only involves an up-regulation of inflammatory mediators as described above but also a constriction of the spiral arteries with consequent hypoxia in the zones closest to the uterine lumen. The precise nature of the endometrial vasoconstrictor involved has yet to be established. Candidates include prostaglandins and other locally produced agents, such as endothelins and angiotensin II. Differences in the ratio of the endothelin receptors (ET_A _and ET_B_) mRNA levels have been reported across the menstrual cycle. The functional relevance of these observations is however unknown [73]. Cyclical changes of angiotensin II and the endometrial expression of the angiotensin receptor subtypes implicate a role in the control of the uterine vasculature [74].

Two phases of menstruation have been proposed [75]. The early events involved in the onset of menstruation, are vasoconstriction and local cytokine changes, initiated by progesterone withdrawal and are likely to be reversible. The subsequent events however, which include the activation of lytic mechanisms are inevitable, implying that this latter phase is progesterone independent and involves cells which may not express PR such as uterine leukocytes and epithelial cells. In support of this proposal, progesterone "add back" in the first 36 hours following progesterone withdrawal prevented menstruation, while progesterone "add back" after 36 hours failed to prevent menses. This observation is expanded in the companion paper in this symposium (Slayden and Brenner) that defines a "critical period" of progesterone withdrawal in the rhesus monkey [76]. This observation is consistent with the view that only the early events, which occur in PR-positive cells, may be inhibited by progesterone "add back".

Amongst the local mediators stimulated by hypoxia, VEGF is a prominent angiogenic factor in the human endometrium [77]. Hypoxia up-regulates VEGF transcription and the VEGF promoter has the response element for hypoxia inducible factor [78,79]. The KDR promoter however lacks the response element for hypoxia inducible factor and hypoxia is therefore unlikely to directly up-regulate KDR. VEGF does however directly up-regulate KDR in vascular endothelium in cerebral brain cultures [80].

There is evidence that experimental hypoxia increases levels of VEGF in non-decidualised and decidualised endometrial stromal cells. Interestingly cAMP, which increases levels of VEGF in endometrial cells, has an additive effect on VEGF production in the presence of hypoxia [81].

Therefore, VEGF, KDR and MMPs are co-ordinately expressed by stromal cells in the upper functional layers of premenstrual stage endometrium at the time of progesterone withdrawal. Since there is evidence that VEGF can upregulate MMPs [82,83], we concluded that a VEGF-KDR-MMP link might be a component of the premenstrual/menstrual process [3].

**Figure 2:**
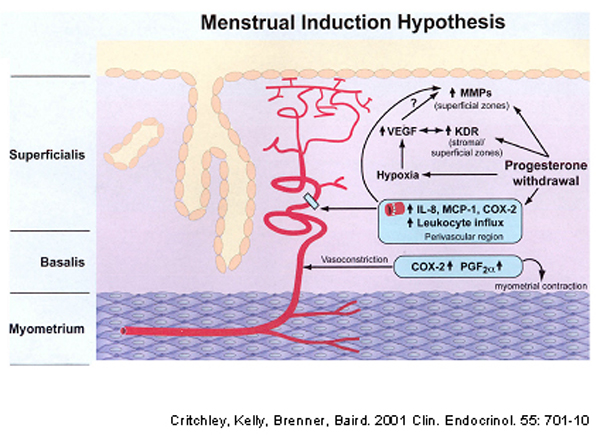
Coincident events of progesterone withdrawal and hypoxia. Progesterone withdrawal results in an up-regulation of inflammatory mediators, production of MMPs, a leukocyte influx and expression of stromal KDR in the upper endometrial zones. There is coincident hypoxia and an up-regulation of VEGF. VEGF binds to its type 2 receptor, KDR and there is a paracrine/autocrine action on the up-regulation of MMP production in the same endometrial upper zone stromal cells. Menstrual sloughing takes place from the superficial regions of the endometrium. Reproduced with permission.

Our working hypothesis of menstrual induction is represented in figure 2; [reproduced from Critchley et al 2001 [3], with permission]. With the demise of the corpus luteum, progesterone levels decline. The consequence of progesterone withdrawal, in cells that contain progesterone receptor, is that inflammatory mediators and locally produced prostaglandins are elevated. These early events in the menstrual process lead to vasoconstriction and cytokine up-regulation. Coincidental constriction of the spiral arteries results in the uppermost endometrial layer becoming hypoxic and the distal zones ischemic. Distal ischemia/hypoxia induces VEGF and VEGF may induce its own receptor KDR within the stromal cells of the uppermost layers. VEGF is able to interact with its receptor KDR and augment, in either a paracrine or autocrine manner, MMP expression by stromal cells. The inflammatory mediators induce a large influx of leukocytes. The combined increase in proteolytic enzymes produced first by stromal cells and later by leukocytic cells results in the characteristic tissue destruction and bleeding associated with menstruation [3,84].

The complexity inherent in the cascading network of menstrual interacting factors means that extensive research is still needed to understand the molecular and cellular processes underlying and controlling these events. Hopefully, as our knowledge increases, we will one day be able to alleviate the severe bleeding problems so many women endure.

## References

[B1] Markee JE (1940). Menstruation in intraocular transplants in the rhesus monkey. Contributions to Embryology of the Carnegie Institution.

[B2] Corner GW, Allen WM (1929). Physiology of the corpus luteum. II. Production of a special uterine reaction (progestational proliferation) by extracts of the corpus luteum. Am J Physiol.

[B3] Critchley HOD, Kelly RW, Brenner RM, Baird DT (2001). The endocrinology of menstruation-a role for the immune system. Clinical Endocrinology.

[B4] Jabbour HN, Kelly RW, Fraser H, Critchley HOD (2006). Endocrine regulation of menstruation. Endocrine Reviews.

[B5] Kao LC, Tulac S, Lobo S, Imani B, Yang JP, Germeyer A, Osteen K, Taylor RN, Lessey BA, Giudice LC (2002). Global gene profiling in human endometrium during window of implantation. Endocrinology.

[B6] Talbi S, Hamilton AE, Vo KC, Tulac S, Overgaard MT, Dosiou C, Le Shay N, Nezhat CN, Kempson R, Lessey BA, Nayak NR, Giudice LC (2006). Molecular phenotyping of human endometrium distinguishes menstrual cycle phases and underlying biological processes in normo-ovulatory women. Endocrinology.

[B7] Riesewijk A, Martin J, van Os R, Horcajadas JA, Polman J, Pellicer A, Mosselman S, Simon C (2003). Gene expression in profiling of human endometrial receptivity on days LH+2 versus LH+7 by microarray technology. Mol Hum Reprod.

[B8] Borthwick JM, Charnock-Jones DS, Tom BD, Hull ML, Teirney R, Phillips SC, Smith SK (2003). Determination of the transcript profile of human endometrium. Mol Hum Reprod.

[B9] Ponnampalam AP, Weston GC, Trajstam AC, Susil B, Rogers PA (2004). Molecular classification of human endometrial cycle stages by transcriptional profiling. Mol Hum Reprod.

[B10] Short RV, Austin CR, Short RV (1984). Oestrous and menstrual cycles. Hormonal Control of Reproduction.

[B11] Baird DT (2000). Overview of advances in contraception. Br Med Bull.

[B12] Noyes RW, Hertig AT, Rock J (1950). Dating the endometrial sample. Fertil Steril.

[B13] Garcia E, Bouchard P, DeBrux J, Berdah J, Frydman R, Schaison G, Milgrom E, Perrot-Applanat M (1988). Use of immunocytochemistry of progesterone and estrogen receptors for endometrial dating. J Clin Endocrinol Metab.

[B14] Lessey BA, Killam AP, Metzger DA, Haney AF, Greene GL, McCarty KS (1988). Immunohistochemical analysis of human uterine estrogen and progesterone receptors throughout the menstrual cycle. J Clin Endocrinol Metab.

[B15] Snijders MP, de Goeij AFPM, Debets-Te Baerts MJC, Rousch MJ, Koudstaal J, Bosman FT (1992). Immunocytochemical analysis of oestrogen receptors and progesterone receptors in the human uterus throughout the menstrual cycle and after the menopause. J Reprod Fertil.

[B16] Critchley HO, Brenner RM, Henderson TA, Williams K, Nayak NR, Slayden OD, Millar MR, Saunders PT (2001). Estrogen receptor beta, but not estrogen receptor alpha, is present in the vascular endothelium of the human and nonhuman primate endometrium. J Clin Endocrinol Metab.

[B17] Slayden OD, Nayak NR, Burton KA, Chwalisz K, Cameron ST, Critchley HOD, Baird DT, Brenner RM (2001). Progesterone antagonists increase androgen receptor expression in the rhesus macaque and human endometrium. J Clin Endocrinol Metab.

[B18] Clark CL, Sutherland RL (1990). Progestin regulation of cellular proliferation. Endocrine Reviews.

[B19] Conneely OM, Lydon JP (2000). Progesterone receptors in reproduction: functional impact of the A and B isoforms. Steroids.

[B20] Wang H, Critchley HOD, Kely RW, Shen D, Baird DT (1998). Progesterone receptor subtype B is differentially regulated in human endometrial stroma. Mol Hum Reprod.

[B21] Brosens JJ, Hayashi N, White JO (1999). Progesterone receptor regulates decidual prolactin expression in differentiating human endometrial stromal cells. Endocrinol.

[B22] Perrot-Applanat M, Deng M, Fernandez H, Lelaidier C, Meduri G, Bouchard P (1994). Immunohistochemical localisation of estradiol and progesterone receptors in human uterus throughout pregnancy: expression in endometrial blood vessels. J Clin Endocrinol Metab.

[B23] Brenner RM, Slayden OD (2004). Steroid receptors in blood vessels of the rhesus macaque endometrium: a review. Arch Histol Cytol.

[B24] Ferenczy A, Gelfand MM, Tzipris F (1983). The cytodynamics of endometrial hyperplasia and carcinoma. A review. Ann Pathol.

[B25] Smith SK (2001). Regulation of angiogenesis in the endometrium. Trends Endocrinol Metab.

[B26] Meduri G, Bausero P, Perrot-Applanat M (2000). Expression of vascular endothelial growth factor receptors in the human endometrium: modulation during the menstrual cycle. Biol Reprod.

[B27] Teng CT, Gladwell W, Beard C, Walmer D, Teng CS, Brenner RM (2002). Lactoferrin gene expression is estrogen responsive in human and rhesus monkey endometrium. Mol Hum Reprod.

[B28] Chauchereau A, Savouret JF, Milgrom E (1992). Control of biosynthesis and post-transcriptional modification of progesterone receptor. Biol Reprod.

[B29] Savouret JF, Bailly A, Misrahi M, Rauch C, Redeuilh G, Chauchereau A, Milgrom E (1991). Characterizartion of the hormone responsive element involved in the regulation of the progesterone receptor gene. EMBO J.

[B30] Shifren JL, Tseng JF, Zaloudek CJ<, Ryan IP, Meng YG, Ferrara N, Jaffe RB, Taylor RN (1996). Ovarian steroid regulation of vascular endothelial growth factor in the human endometrium: implications for angiogenesis during the menstrual cycle and in the pathogenesis of endometriosis. J Clin Endocrinol Metab.

[B31] Green S, Walter P, Kumar V, Krust A, Bornert JM, Argos P, Chambon P (1986). Humasn oestrogen receptor cDNA: sequence, expression and homology to v-erb-A. Nature.

[B32] Kuiper GG, Enmark E, Pelto-huikko M, Nilsson S, Gustafsson JA (1996). Cloning of a novel receptor expressed in rat prostate and ovary. Proc Natl Acad Sci USA.

[B33] Enmark E, Pelto-Huikko M, Grandien K, Lagercrantz S, Lagercrantz J, Fried G, Nordenskjold M, Gustafsson JA (1997). Human estrogen receptor β-gene structure, chromosomal localization, and expressive pattern. J Clin Endocrinol Metab.

[B34] Brenner RM, West NB, McClellan MC, Hild-Petito SA, Stouffer RL, Gustafsson JA, Eriksson H, Carlsted-Duke J (1998). Cellular localization of estrogen and progestin receptors in the macaque reproductive system. The Steroid/Thyroid Hormone Receptor Family and Gene Regulation.

[B35] Leece G, Meduri G, Ancelin M, Bergeron C, Perrot-Applanat (2001). Presence of estrogen receptor β in the human endometrium through the cycle: expression in glandular, stromal and vascular cells. J Clin Endocrinol Metab.

[B36] Critchley HO, Henderson TA, Kelly RW, Scobie GS, Evans LR, Groome NP, Saunders PT (2002). Wild-type estrogen receptor (ERbeta1)and the splice variant (ERbetacx/beta2) are both expressed within the human endometrium. J Clin Endocrinol Metab.

[B37] Cowley SM, Hoare S, Mosselman S, Parker SG (1997). Estrogen receptors α and β form heterodimers on DNA. J Biol Chem.

[B38] Petterson K, Grandien K, Kuiper GGJM, Gustafsson JA (1997). Mouse estrogen receptor β forms estrogen receptor response element-binding heterodimers with estrogen receptor α. Mol Endocrinol.

[B39] Paech K, Webb P, Kuiper GGJM, Nilsson S, Gustafsson JA, Kushner PJ, Scanlan TS (1997). Differential ligand activation of estrogen receptors ERα and ERβ at AP1 sites. Science.

[B40] Hall JM, McDonnell DP (1999). The estrogen receptor β-isoform (ERβ) of the human estrogen receptor modulates ERα transcriptional activity and is a key regulator of the cellular response to estrogens and antiestrogens. Endocrinology.

[B41] Ogawa S, Inoue S, Watanbe T, Hiroi H, Orimo A, Hosoi T, Ouchi Y, Muramatsu M (1998). The complete primary structure of human estrogen receptor β (hERβ) and its heterodimerization with ERα in vivo and in vitro. Biochem Biophys Res Commun.

[B42] Nilsson S, Makela S, Treuter E, Tujague M, Thomsen J, Andersson G, Enmark E, Pettersson K, Warner M, Gustafsson JA (2001). Mechanisms of estrogen action. Physiol Rev.

[B43] Okon MA, Laird SM, Tuckerman EM, Li TC (1998). Serum androgen levels in women who have recurrent miscarriages and their correlation with markers of endometrial function. Fertil Steril.

[B44] Hirst JJ, Slayden OD, West NB, Brenner RM (1992). Androgens suppres estrogen and progestin receptors in the endometrium and oviduct of rhesus monkeys. The Society for Gynaecological Investigation Program.

[B45] Burton KA, Henderson Ta, Hiller SG, Mason JI, Habib F, Brenner RM, Critchley HO (2003). Local levonorgestrel regulation of androgen receptor and 17beta-hydroxysteroid dehydrogenase type 2 expression in human endometrium. Hum Reprod.

[B46] Narvekar N, Cameron S, Critchley HO, Lin S, Cheng L, Baird DT (2004). Low-dose mifepristone inhibits endometrial proliferation and up-regulates androgen receptor. J Clin Endocrinol Metab.

[B47] Brenner RM, Slayden OD, Critchley HO (2002). Anti-proliferative effects of progesterone antagonists in the primate endometrium: a potential role for the androgen receptor. Reproduction.

[B48] Brenner RM, Slayden OD, Nayak N, Baird DT, Critchley HOD (2003). A role for the androgen receptor in the endometrial proliferative effects of progesterone antagonists. Steroids.

[B49] Slayden OD, Brenner RM (2003). Flutamide counteracts the antiproliferative effects of antiprogestins in the primate endometrium. J Clin Endocrinol Metab.

[B50] Hodgen GD, van Uem JF, Chillik CF, Danforth DR, Wolf JP, Neulen J, Williams RF, Chwalisz K (1994). Non-competitive ant-oestrogenic activity of progesterone antagonists in primate models. Hum Reprod.

[B51] Peltoketo EH, Luu-The V, Simard J, Adamski J (1999). 17β hydroxysteroid dehydrogenase (HSD)/17-hetoreductase (KSR) family; nomenclature and main characteristics of the 17HSD/KSR enzymes. J Mol Endocrinol.

[B52] Labrie F, Luu-The V, Lin XS, Simard J, Labrie C, El-Alfy M, Pelletier G, Belanger A (2000). Intracrinology: role of the family of 17β hydroxysteroid dehydrogenases in human physiology and disease. J Mol Endocrinol.

[B53] Casey ML, McDonald PC, Andersson S (1994). 17β-Hydroxysteroid dehydrogenase type 2: chromosomal assignment and progestin regulation of gene expression in human endometrium. J Clin Invest.

[B54] Mustonen MV, Isomaa VV, Vaskivuo T, Tapanainen J, Poutanen MH, Stenback F, Vihko RK, Vihko PT (1998). Human 17β-hydroxysteroid dehydrogenase type 2 messenger ribonucleic acid expression and localization in tem placenta and in endometrium during the menstrual cycle. J Clin Endocrinol Metab.

[B55] Maentausta O, Svalander P, Danielsson KG, Bygdeman M, Vihko R (1993). The effects of an antiprogestin, mifepristone, and an antiestrogen, tamoxifen, on endometrial 17β-hydroxysteroid dehydrogenase and progestin and estrogen receptors during the luteal phase of the menstrual cycle: an immunohistochemical study. J Clin Endocrinol Metab.

[B56] Kloosterboer HJ, Vonk-Noordegraaf CA, Turpijn EW (1988). Selectivity in progesterone and androgen receptor binding of prostagens used in oral contraceptives. Contraception.

[B57] Lemus AE, Vilchis F, Damsky R, Chavez BA, Garcia GA, Grillasca I, Perez-Palacios G (1992). Mechanism of action of levonorgestrel: in vitro metabolism and specific interactions with steroid receptors in target organs. J Steroid Biochem Mol Biol.

[B58] Nilsson CJ, Luukainen T, Arko H (1978). Endometrial morphology of women using a D-norgestrel-releasing intrauterine device. Fertil Steril.

[B59] Silverberg SG, Haukkamaa M, Arko H, Nilsson CG, Luukkainen T (1986). Endometrial morphology during long-term use of levonorgestrel-releasing intrauterine devices. Int J Gynecol Pathol.

[B60] Critchley HO, Wang H, Kelly RW, Gebbie AE, Glasier AF (1998). Progestin receptor isoforms and prostaglandin dehydrogenase in the endometrium of women using a levonorgestrel-releasing intrauterine system. Hum Reprod.

[B61] Lockwood CJ, Krikun G, Hausknecht VA, Papp C, Schatz F (1998). Matrix metalloproteinase and matrix metalloproteinase inhibitor expression in endometrial stromal cells during progestin-initiated decidualization and menstruation-related progestin withdrawal. Endocrinology.

[B62] Schatz F, Papp C, Aignewr S, Krikun G, Hausknecht V, Lockwood CJ (1997). Biological mechanisms underlying the clinical effects of RU486: modulationof cultured endometrial stromal cell stromelysin-1 and prolactin expression. J Clin Endocrinol Metab.

[B63] Salamonsen LA, Woolley DE (1996). Matrix metalloproteinases in normal menstruation. Hum Reprod.

[B64] Salamonsen LA, Zhang J, Hampton A, Lathbury L (2000). Regulation of matrix metalloproteinases in human endometrium. Hum Reprod.

[B65] Rudolph-Owen LA, Slayden O, Matrisan LM, Brenner RM (1998). matrix metalloproteinase expression in Macaca mulatto endometrium: evidence for zone-specific regulatory tissue gradients. Biol Reprod.

[B66] Swahn ML, Bygdeman M, Cekan S, Xing S, Masironi B, Johanisson E (1990). The effect of RU486 administered during the early luteal phase on bleeding, hormonal parameters, and endometrium. Hum Reprod.

[B67] Spitz IM, Bardin CW (1993). Mifepristone (RU486) – a modulator of progestin and glucocorticoid action. New Eng J Med.

[B68] Irwin JC, Kirk D, Gwatkin RBL, Navre M, Cannon P, Giudice LC (1996). Human endometrial matrix metalloproteinase-2, a putative menstrual proteinase. Hormonal regulation in cultured stromal cells and messenger RNA expression during the menstrual cycle. J Clin Invest.

[B69] Schatz F, Aigner S, Papp C, Toth-Pal E, Hausknecht V, Lockwood CJ (1995). Plasminogen activator activity during decidualisation of human endometrial cells is regulated by plasminogen activator inhibitor 1. J Clin Endocrinol Metab.

[B70] Baird DT, Cameron St, Critchley HO, Drudy Ta, Howe A, Jones RL, Lea RG, Kelly RW (1996). Prostaglandins and menstruation. Eur J Obstet Gynecol Reprod Biol.

[B71] Tabibzadeh S (1996). The signals and molecular pathways involved in human menstruation, a unique process of tissue destruction and remodelling. Mol Hum Reprod.

[B72] Nayak NR, Critchley HOD, Slayden O, Menrad A, Chwalisz K, Baird DT, Brenner RM (2000). Progesterone withdrawal up-regulates vascular endothelial growth factor receptor type 2 in the superficial zone stroma of the human and macaque endometrium: potential relevance to menstruation. J Clin Endocrinol Metab.

[B73] O'Reilly G, Charnock-Jones DS, Davenport AT, Cameron IT, Smith SK (1992). Presence of messenger ribonucleic acid for endothelin-1, endothelin-2, and endothelin-3 inhuman endometrium and a change in the ratio of ET-A and ET-B receptor subtype across the menstrual cycle. J Clin Endocrinol Metab.

[B74] Ahmed A, Li X-F, Shams M, Gregory J, Rollason T, Barnes NM, Newton JR (1995). Localization of the angiotensin II and its receptor subtyope expression inhuman endometrium and identification of a novel high-affinity angiotensin II binding site. J Clin Invest.

[B75] Kelly RW, King AE, Critchley HO (2001). Cytokine control in human endometrium. Reproduction.

[B76] Slayden OD, Mah K, Brenner RM (1999). A critical period of progesterone withdrawal exists for endometrial MMPs and menstruation in macaques. Biol Reprod.

[B77] Sharkey AM, Day K, McPherson A, Malik S, Licence D, Smith SK, Charnock-Jones DS (2000). Vascular endothelial growth factor expression in human endometrium is regulated by hypoxia. J Clin Endocrinol Metab.

[B78] Goldberg MA, Schneider TJ (1994). Similarities between the oxygen-sensing mechanisms regulating the expression of vascular endothelial growth factor and erythropoietin. J Biol Chem.

[B79] Gerber HP, Condorelli F, Park J, Ferrara N (1997). Differential transcriptional regulation of the two vascular endothelial growth factor receptor genes, Flt-1, but not Flt-1/KDR, is up-regulated by hypoxia. J Biol Chem.

[B80] Kremer C, Breier G, Risau W, Plate KH (1997). Up-regulation of flk-1/vascular endothelial growth factor receptor 2 by its ligand in a cerebral slice culture system. Cancer Res.

[B81] Popovici RM, Irwin JC, Giaccia AJ, Giudice LC (1999). Hypoxia and cAMP stimulate vascular endothelial stromal cells: potential relevance to menstruation and endometrial regeneration. J Clin Endocrinol Metab.

[B82] Wang H, Keiser JA (1998). Vascular endothelial growth factor upregulates the expression of matrix metalloproteinases in vascular smooth muscle cells: role of flt-1. Circ Res.

[B83] Unemori EN, Ferrara N, Bauer EA, Amento EP (1992). Vascular endothelial growth factor induces interstitial collagenase expression in human endothelial cells. J Cell Physiol.

[B84] Critchley HOD, Kelly RW, Brenner RM, Baird DT (2003). Antiprogestins as a model for progesterone withdrawal. Steroids.

